# Moving from minimally invasive to totally endoscopic mitral valve surgery: a retrospective review of outcomes

**DOI:** 10.1093/icvts/ivaf106

**Published:** 2025-05-09

**Authors:** Mark Boyle, Ahmed Elfadil, Saeed Mirsadraee, Toufan Bahrami

**Affiliations:** Department of Surgery and Cancer, Imperial College, London, UK; Department of Cardiothoracic Surgery, Royal Victoria Hospital, Belfast, UK; Department of Cardiac Surgery, Royal Brompton and Harefield Hospitals, London, UK; Department of Radiology, Royal Brompton and Harefield Hospitals, London, UK; Department of Cardiac Surgery, Royal Brompton and Harefield Hospitals, London, UK

**Keywords:** minimally invasive, valve surgery, totally endoscopic

## Abstract

**OBJECTIVES:**

Mitral valve surgery has undergone significant advancements with the emergence of minimally invasive mitral surgery harnessing endoscopic technology to facilitate repair through a right anterior thoracotomy. Further refinement within the field has borne the novel totally endoscopic approach, reducing incision size, and surgical trauma, to 3 cm. While there is evidence to support non-inferiority of minimally invasive techniques compared to a traditional sternotomy, a knowledge gap exists regarding the comparative safety and efficacy between minimally invasive modalities, necessitating.

**METHODS:**

A retrospective review of outcomes following totally endoscopic and minimally invasive mitral valve surgery by right anterior thoracotomy was completed. One hundred eighty-six patients were included, all operations having been performed by a single surgeon, between January 2019 and June 2023. The hypothesis posits that the former offers an equivalence in repair while reducing postoperative pain, bleeding from the wound and enhancing cosmesis.

**RESULTS:**

While low 30-day mortality rates were seen in both cohorts, the totally endoscopic group exhibited lower rates of patients discharged with opiates (16% vs 23%), reduced blood product transfusion requirements (33% vs 43%) and shorter postoperative stays (mean of 9.2 days vs 11.4 days).

**CONCLUSIONS:**

Moving from minimally invasive to totally endoscopic mitral valve surgery has been a positive experience with key patient advantages characterized by smaller incisions and avoidance of rib spreading. In this dataset, improved patient outcomes such as postoperative bleeding, pain, length of hospital stay and cosmesis were observed with all limitations given its fully uncontrolled nature. Validation of these findings warrants a larger study.

## Graphical Abstract

**Figure:**
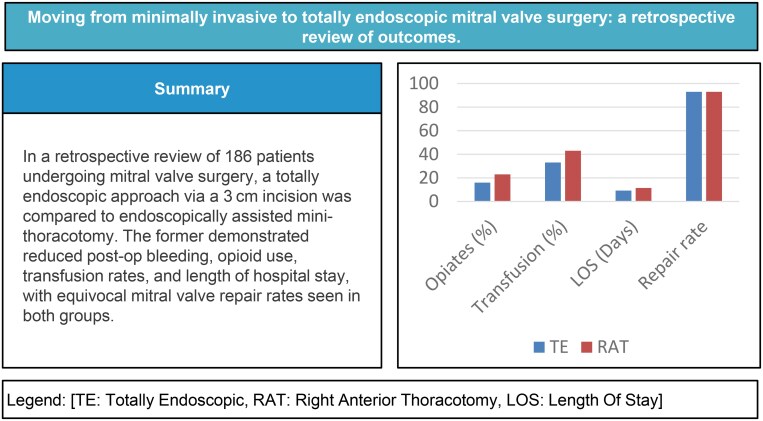

## INTRODUCTION

Mitral valve disease is a global issue with mitral regurgitation (MR) having a quoted incidence in the region of 24.2 million and being directly attributable for 34 000 deaths in 2019 [1]. Despite rapid advancement of innovative transcatheter modalities, surgical repair remains the treatment of choice for mitral disease [2]. Driven by changing patient demographics and expanding technological capabilities, there have been major advancements in the field of mitral valve surgery. Supported by a body of research demonstrating its surgical efficacy comparable to conventional sternotomy, minimally invasive mitral valve surgery (MIMVS) is now becoming increasingly routine [3–5]. Furthermore, comparable medium-term outcomes have now been demonstrated in the minimally invasive surgical repair of greater complexity mitral valve disease [6].

Beyond its equivalency, MIMVS offers an array of advantages, including reduced surgical trauma, expedited recovery, enhanced cosmetic outcomes and shorter hospital stays, which have garnered an increasing number of advocates for its implementation as the standard of care [7]. However, widespread adoption has been slow, with lingering concern that a durable repair is compromised for the sake of a smaller incision [8].

In this series, we describe our experience, moving from minimally invasive—endoscopically assisted—mitral valve surgery to the more novel, totally endoscopic mitral valve surgery.

### Totally endoscopic mitral valve surgery

All patients undergo a computed tomography (CT) aortogram with 3D reconstruction as standard, prior to consideration of a minimally invasive mitral valve procedure. This investigation is preformed specifically to assess calcification of the ascending aorta, atheroma of the abdominal aorta and the size and tortuosity of the femoral vessels. All cases are then discussed in a mitral multi-disciplinary team (MDT) meeting before a patient is offered a minimally invasive mitral operation.

Anaesthesia is induced with a double-lumen tube used to permit single, left lung ventilation. The patient is placed supinely on the operating table, external defibrillator pads are placed appropriately, and the right hemithorax is raised slightly. Draping is performed in a routine manner ensuring adequate exposure of the right chest, sternum and both groins with considerations for two pneumatic holding arms (Unitrac, Aesculap, B. Braun, Germany) attached to the operating table at the head end. Transoesophageal echocardiography (TOE) is prepared prior to being used intra-operatively including to guide cannula placement, assess the valve and the repair.

Peripheral extracorporeal circulation is established via the femoral vessels. Cardiopulmonary bypass (CPB) is achieved by way of femoral arterial and venous cannulation and direct transthoracic aortic clamping. The arterial and venous cannulae are placed in the right femoral artery and vein and maneuvered into place using a Seldinger technique under TOE guidance. After vacuum-assisted CPB is established, with up to 60 mmHg of negative pressure being used to ensure the right atrium is clear of blood, the patient is then cooled to 32°C.

**Figure 1:**
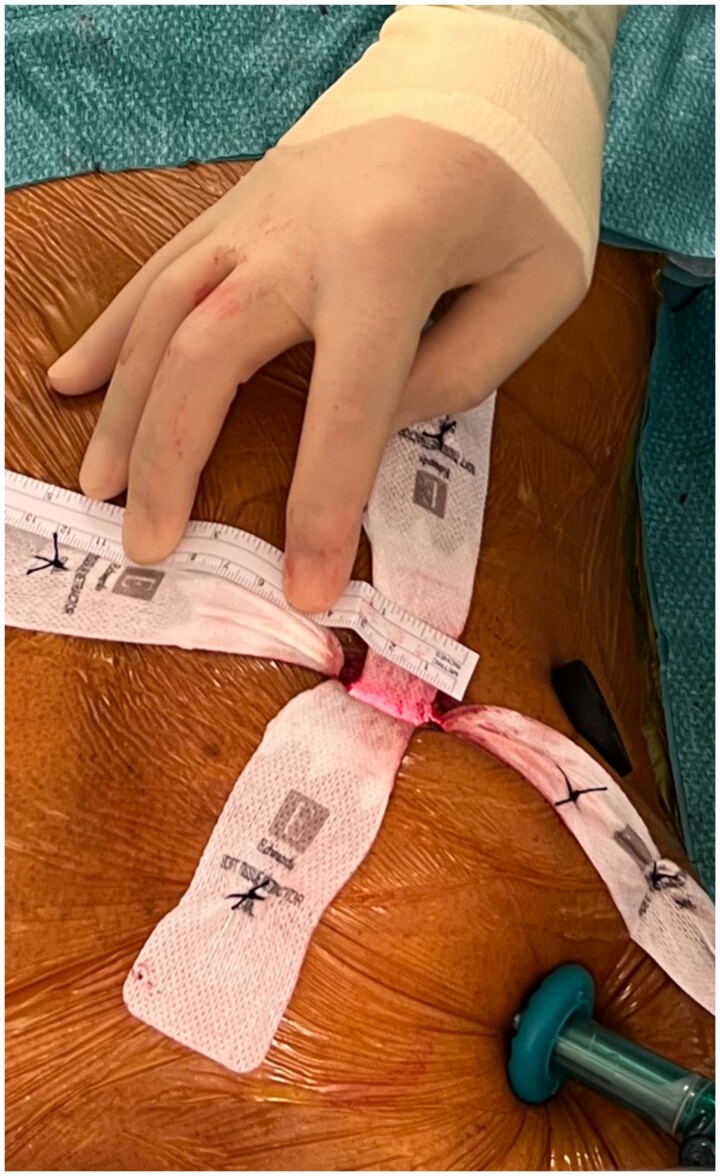
Intra-operative photo demonstrating the size of peri-areolar incision (3 cm).

**Figure 2:**
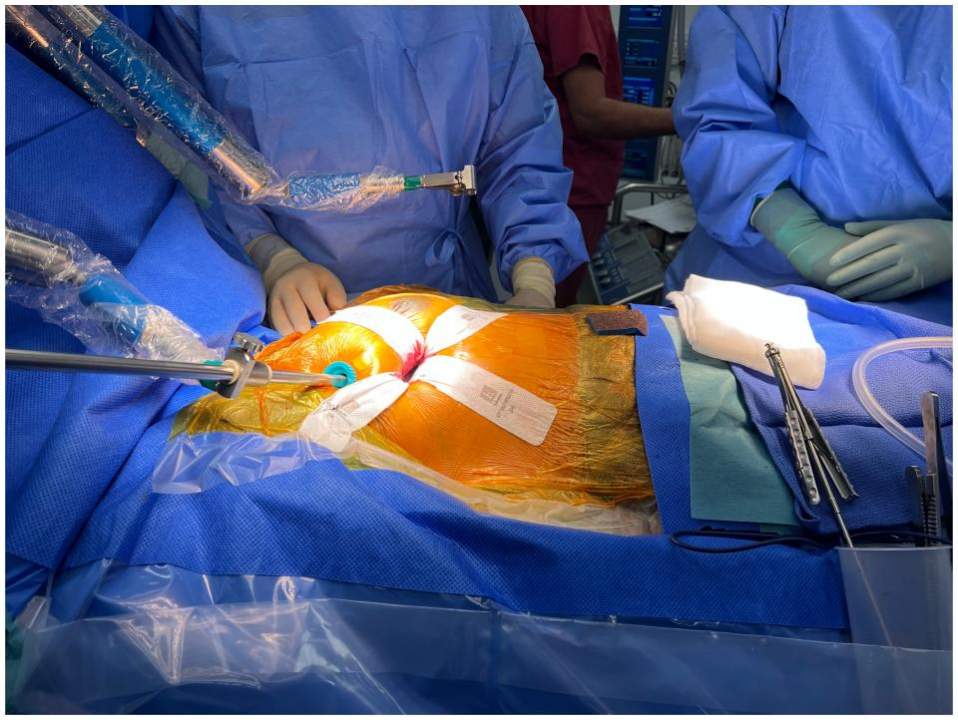
Intra-operative photo demonstrating the operating surgeons view, with the camera system held in place by a Unitrac pneumatic arm, and an XS soft tissue retractor in place via the 3 cm peri-areolar incision.

Totally endoscopic mitral valve surgery is performed via a small 3 cm curved incision along the right areolar border (see Fig. 1). This incision facilitates access to the heart via the fourth or fifth intercostal space with no need for a larger incision, undermining of the incision or a rib retractor. Following the incision, a soft tissue retractor (size: extra-small [XS]) is placed within the intercostal space and is secured with stay sutures, see Fig. 2. Two 11 mm port sites are inserted in the thorax in a parasagittal line (between the right midclavicular and anterior axillary line) one rib space above and below the peri-areolar incision. The superior ports permit introduction of a CO_2_ insufflator, and the thoracoscope (EinsteinVision 3.0 3D Camera system, Aesculap, B. Braun, Germany) which is held in place by the pneumatic arm, and feeds into a 32’ full HD 3D monitor (EinsteinVision 3.0 3D Camera system, Aesculap, B. Braun, Germany). A fourth incision is made in the mid axillary line at the axial level of the peri-areolar incision to facilitate the aortic cross-clamp and cardioplegia line.

**Figure 3:**
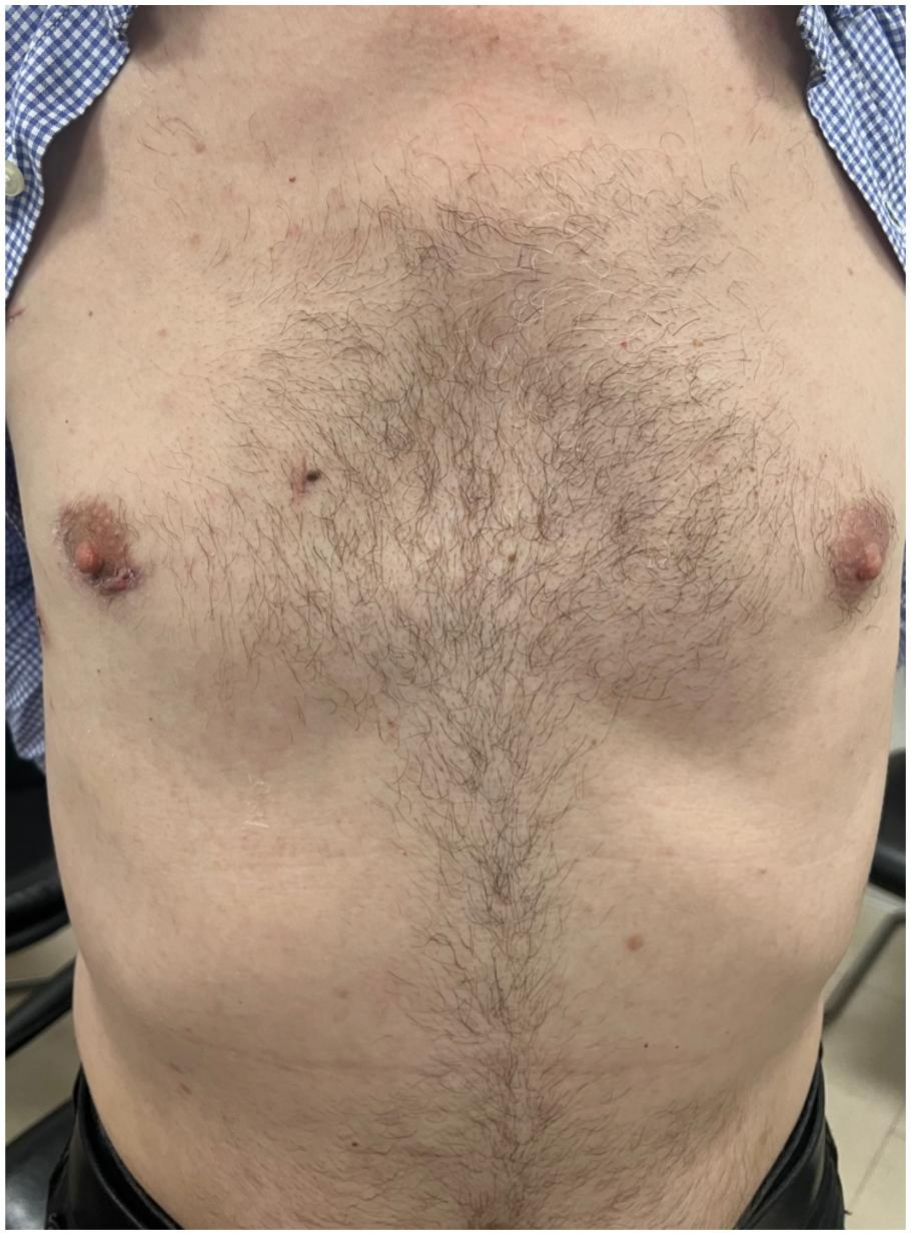
Cosmetic result following a totally endoscopic mitral valve repair.

The ascending aorta is clamped using a Chitwood transthoracic aortic cross-clamp (Scanlan International Inc, Minneapolis, MN), and antegrade blood cardioplegia is delivered directly into the ascending aorta via a DLP (Medtronic Ltd, UK) catheter. The mitral valve is approached in the typical fashion with dissection of Sondergaards groove; the incision is then extended superiorly towards the left atrial roof. A foldable ‘HV’ atrial retractor (USB Medical Ltd, Philadelphia, USA) is then placed in an intra-atrial position and held in place by the second pneumatic arm. The atrial retractor holder is inserted through an additional parasternal port. Once the left atrium is fully opened and the mitral valve accessible, the lesions are assessed and then the appropriate reconstructive technique is selected. These mitral valve procedures are performed under total endoscopic guidance using specialist ‘Valvegate Pro’ endoscopic cardiac surgery instruments (Emmat Medical Ltd, UK). All patients receive an intraoperative TOE before and after weaning from CPB machine. Paravertebral blocks (PVBs) are performed under endoscopic guidance to provide postoperative analgesia. Outpatient review of a patient’s healed wounds from a totally endoscopic mitral valve repair is seen in Fig. 3.

### Endoscopically assisted mitral valve surgery

Endoscopically assisted surgery differs in several key aspects. A right-sided mini-thoracotomy of 6–8 cm is made in the fourth or fifth intercostal space in the infra-mammary groove. Following knife to skin, the subcutaneous tissue is divided with diathermy. Dissecting close to the upper edge of the rib while dividing the external, internal and innermost intercostal muscles, this dissection is extended to >10 cm (IE larger than the skin incision). The reasons for this are to reduce tension on the ribs during rib retraction and subsequently mitigate risk of rib fracture. Once the pleural cavity is entered, a rib retractor is used to facilitate adequate access of instruments. Surgery on the mitral valve is performed in a similar fashion as the totally endoscopic approach, but notably, under direct vision through the thoracotomy site with video assistance through an additional port, rather than relying entirely on the visual from a thoracoscope. Closure of the thoracotomy site typically requires the use of Ethibond 5 pericostal sutures, or in some instances, transcostal suture. However, this can cause significant postoperative pain due to binding of the ribs, and by association, compression of the neurovascular bundle.

### Data availability and ethics statement

This is a review of outcomes for two operative approaches indicated in the treatment of severe MR. This study was conducted in accordance with the ethical principles outlined in the WMA Declaration of Helsinki and the WMA Declaration of Taipei regarding the collection, storage and use of research data. Ethics committee approval was waived, as the study was retrospective in nature and conducted as part of a service evaluation approved through the NHS Trust audit processes.

All patient data were anonymized and securely stored in compliance with institutional policies and General Data Protection Regulation guidelines. No biological material was collected or stored as part of this study. All patients provided their written informed consent for the use of their data and images to be used for research and publication purposes.

## METHODS

A retrospective review of clinical outcomes for two MIMVS approaches was completed. All operations were performed by one Consultant Cardiac Surgeon (T.B.), operating at two sites in one UK NHS trust between January 2019 and June 2023. The patients were divided into two cohorts, those who underwent endoscopically assisted mitral valve surgery, by way of a right anterior mini-thoracotomy, and those who underwent totally endoscopic mitral valve surgery. Patients who had a planned sternotomy and those who also had attention to their aortic valve or coronary artery bypass grafting were excluded. Patients who underwent standard concomitant treatments such as attention to their tricuspid valve, atrial appendage occlusion, patient foramen ovale (PFO) closure or ablation were included. All patients underwent CT angiography prior to each procedure for procedural and bypass feasibility assessment. Redo procedures and patients who had mitral valve surgery for infective endocarditis were also eligible for inclusion in this study, provided, they underwent either of the aforementioned minimally invasive approaches.

Key intraoperative data points were extracted, including CPB time, aortic cross-clamp time, the surgeon’s assessment of the valve and the method of repair or replacement.

All data on 30-day complications were collected in accordance with a predefined list, including 30-day mortality, conversion to sternotomy, myocardial infarction, stroke, renal injury according to Acute Kidney Injury Network (AKIN) class II-III, the necessity for surgical reintervention (e.g. haemothorax, pneumothorax, empyema or failed repair), prolonged ventilation exceeding 24 hours and the requirement for inotropes for more than 24 hours. Additionally, morbidity indicators such as infection, cerebrovascular accident (CVA), length of hospital stay and discharge with opiates were logged.

Continuous variables are presented as mean ± standard deviation, and categorical variables are expressed as a percentage of the total.

## RESULTS

During the study interval, 186 patients met the inclusion criteria having underwent a minimally invasive mitral valve operation. Overall, 131 patients had their operation performed via a right mini-thoracotomy in an endoscopically assisted fashion. Fifty-five patients underwent a totally endoscopic approach via a peri-areolar incision.

Baseline demographics and comorbidity profiles are summarized in Table 1. In the totally endoscopic group, patients were more likely to be younger, female and less likely to have diabetes, hypertension or a smoking history. Conversely, the totally endoscopic group had an increased mean EUROSCORE II and were more likely to have existing atrial fibrillation (AF). The baseline preoperative ejection fractions, renal function and body mass index (BMI) were similar in both groups.

**Table 1:** Baseline characteristics

		Totally endoscopic (55)	Endoscopically assisted (131)
Age mean ± SD years		58.8 ± 14.0	64.2 ± 11.8
Female		24 (44%)	32 (24%)
BMI mean ± SD, kg/m^2^		25.4 ± 3.8	26.0 ± 4.2
EUROSCORE II mean ± SD		2.3 ± 3.1	2.1 ± 2.1
Diabetes mellitus		1 (2%)	13 (10%)
Ex/current smoker		5 (9%)	40 (27%)
Hypertensive		13 (24%)	61 (47%)
Creatinine		76.9 ± 21.5	84.9 ± 34.1
Renal impairment	Moderate	11 (20%)	48 (36%)
	Severe	2 (4%)	8 (6%)
LVEF		57.5 ± 8.5	57.2 ± 8.7
Atrial fibrillation		15 (27%)	26 (20%)
Pulmonary hypertension *147		13/45 (29%)	38/97 (39%)

BMI: body mass index; LVEF: left ventricular ejection fraction.

* 142 of the patients had recorded data of pulmonary hypertension, the remaining 44 did not have this field completed in the data submission forms.

Operative data are presented in Table 2. In the totally endoscopic group, patients were more likely to be operated on in an urgent or emergent fashion, and they had an increased cumulative CPB time and cross-clamp time. The mitral repair rate was equivocal in both groups at 93%.

**Table 2:** Intraoperative characteristics

	Totally endoscopic (55)	Endoscopically assisted (131)
Elective	45 (82%)	118 (89%)
Urgent	9 (16%)	6 (11%)
Emergent	1 (2%)	0 (-)
Cumulative bypass time mean ± SD, mins	208.9 ± 51.4	162.7 ± 34.4
Cumulative cross-clamp time mean ± SD, mins	127.5 ± 36.5	100.6 ± 26.6
Mitral replacement	12 (22%)	14 (11%)
Mitral repair	43 (78%)	117 (89%)
Mitral repair rate (number of repairs/all those deemed repairable at MDT)	43/46 (93%)	117/126 (93%)

In-hospital outcomes are seen in Table 3. There were no reports of a postoperative transient ischaemic attack (TIA) or a stroke in the totally endoscopic groups, with 3 of the 131 patients in the endoscopically assisted group suffering a neurological event. With respect to arrythmia, the groups were evenly matched in the necessity for pacemaker insertion (4%); however, new postoperative AF (POAF) was lower in the totally endoscopic cohort (18% vs 26%). From a thoracic perspective, the rate of postoperative pneumothoraces requiring drainage (4% vs 3%) and the incidence of pleural effusion requiring drainage (7% vs 8%) were similar. There was no reported acute kidney injury (AKI) in the totally endoscopic group. Prolonged inotropic support (>24 hours) was twice as common in the endoscopically assisted group (15% vs 7%) and prolonged ventilation (>24 hours) three times as common (6% vs 2%). Re-intervention for bleeding was found in 5% of the endoscopically assisted group and 2% were converted to sternotomy; however, there were no instances of either reintervention for bleeding or conversion to sternotomy within the 55 patients who had a totally endoscopic operation.

**Table 3:** In-hospital outcomes

	All (186)	Totally endoscopic (55)	Endoscopically assisted (131)
Delirium	7 (4%)	0 (-)	7 (5%)
TIA	1 (1%)	0 (-)	1 (1%)
Stroke	2 (1%)	0 (-)	2 (2%)
New AF	44 (24%)	10 (18%)	34 (26%)
New pacemaker	7 (4%)	2 (4%)	5 (4%)
Pleural effusion	14 (8%)	4 (7%)	10 (8%)
Pneumothorax	6 (3%)	2 (4%)	4 (3%)
UTI	1 (1%)	0 (-)	1 (1%)
Wound infection	6 (3%)	1 (2%)	5 (4%)
AKI I	3 (2%)	0 (-)	3 (2%)
AKI II-III	3 (2%)	0 (-)	3 (2%)
LCOS >24 hours	24 (13%)	4 (7%)	20 (15%)
Conversion to sternotomy	3 (2%)	0 (-)	3 (2%)
MI	2 (1%)	0 (-)	2 (2%)
Re-intervention for bleeding	7 (4%)	0 (-)	7 (5%)
Prolonged ventilation >24 hours	9 (5%)	1 (2%)	8 (6%)

TIA: transient ischaemic attack; AF: atrial fibrillation; UTI: urinary tract infection; AKI: acute kidney injury; LCOS: low cardiac output state; MI: myocardial infraction.

Key performance indicators for both operations are seen in Table 4. There were no deaths within 30 days for the totally endoscopic cohort, compared to two deaths in the endoscopically assisted group. There were an increased number of patients discharged with opiates in the endoscopically assisted group (23% vs 16%), an increased percentage of patients requiring blood product transfusion (43% vs 33%) and notably an increased length of postoperative stay (mean of 11.4 days vs 9.2 days).

**Table 4:** Key performance indicators of both operations

	All (186)	Totally endoscopic (55)	Endoscopically assisted (131)
30-day mortality	2 (1%)	0 (-)	2 (2%)
Discharge with opiates?	39 (21%)	9 (16%)	30 (23%)
Blood products used?	74 (40%)	18 (33%)	56 (43%)
LOS—Post op	10.8 ± 8.3	9.2 ± 5.3	11.4 ± 9.2

LOS: length of stay.

During the study period, 2 of the 131 endoscopically assisted operations were performed on patients with mitral valve endocarditis, compared to 5 of the 55 totally endoscopic operations. Over half of all operations included concomitant procedures such as PFO closure, AF ablation or attention to the tricuspid valve.

## DISCUSSION

Across both modalities we observed low mortality (1%) and stroke rate (1%). The most frequently occurring postoperative complication encountered was the onset of POAF (24%); however, this rate is consistent with existing literature [9].

One finding of note in this study is the moderate increase in CPB time in the totally endoscopic cohort. Existing literature attests to the requirements for longer CPB time in a minimally invasive surgical approach to the mitral valve due to the increased technical demand [10]. However, a central concern of prolonged CPB time is a sequalae of complications including AKI and stroke with some studies suggesting CPB time as a predictor of increased mortality [11, 12]. A recent randomized control trial (RCT) comparing a mini-thoracotomy to median sternotomy in the treatment of mitral disease found no difference in stroke rates [13]. Importantly, within our study, the moderate increase in bypass time in our totally endoscopic cohort did not correlate with an increase in CVA (0% vs 2%), or significant AKI (0% vs 2%); moreover, the incidence of CVA and AKI in both groups falls below reported figures [14, 15].

The risk of death, wound infection, myocardial infarction and readmission at 30 days has been reported as not statistically different by Xu *et al.* [16] when comparing minimally invasive surgical approaches to established surgical methods. Both cohorts in this study appear to have comparable postoperative outcomes with respect to said data points including incidence of wound infection, myocardial infarction and need for thoracic intervention.

The aetiology of mitral disease in both groups was varied, with 2 of the 131 endoscopically assisted operations performed for mitral valve endocarditis, and 5 of the 55 totally endoscopic cohort. Notably, over half of all patients required concomitant procedures including PFO closure, AF ablation or attention to their tricuspid valve. The majority of patients in both groups proceeded to undergo a mitral valve repair. This is keeping with work undertaken by Gammie and colleagues, who demonstrated as per the Society of Thoracic Surgeons (STS) database, the rate of repair is appreciably higher when patients were operated via a minimally invasive technique (85% vs 67%) [17].

Crucially, the totally endoscopic approach appears to have an advantage in several key areas. Firstly, the reduction in incidence of patients requiring a return to theatre for bleeding. Five percent of cases returned to theatre for bleeding in the endoscopically assisted group compared to 0 cases in the totally endoscopic cohort. Re-exploration for bleeding has been quoted in the literature to be as high as 8% [16]. Significant bleeding requiring return to theatre is not a rare complication following cardiac surgery, but it is accompanied with inferior outcomes [18]. A common cause of this postoperative complication within thoracoscopic surgery is bleeding from the port sites, highlighting the benefit of a smaller incision size and avoidance of further intercostal muscle dissection [19]. In addition to a lower return to theatre rate, there too appears to be a correlated reduction in need for blood product transfusion. Red blood cell transfusion is independently associated with further postoperative morbidity, propagated by the associated risks of immunosuppression, fever, sepsis and transfusion-related lung injury (TRALI) [20], ultimately proving to be deleterious to long-term outcomes [20, 21].

It is accepted that there is superior cosmesis associated with a minimally invasive operation compared to a median sternotomy. Furthermore, the discrete 3 cm wound in the peri-areolar line following a totally endoscopic mitral valve operation contrasts with the larger, more visible, non-anatomical, lateral incision associated with endoscopically assisted techniques, which, as per one study, averages a length of 8.2 ± 1.85 cm [22]. One study using patient self-assessment scores to investigate satisfaction of both aesthetics and sensation of postoperative scars from a peri-areolar incision concluded that this minimally invasive approach delivers patient-satisfying results [23]. Often, cosmesis is discounted as an inconsequential side effect following major surgery. However, several studies have demonstrated the debilitating effects surgical scarring can have on a patient’s body image and the cost it may have on their self-esteem [24]. Moreover, further analysis has identified a disconnect between surgeon and patient on this front, across many surgical specialities [25]. Therefore, more attention must be paid to the patient’s ideas, concerns and expectations to achieve a suitably pragmatic and patient-centred result.

Finally, in our study, a totally endoscopic mitral valve operation resulted in a reduced length of hospital stay and a reduction in the number of patients who required opiate analgesia to take home on discharge. Avoidance of rib spreading and pericostal suturing has been suggested as the basis for this improvement in the more novel, totally endoscopic approach.

### Limitations of the study

The limitations of this study include the single-surgeon, retrospective design of the study and paucity of long-term follow-up. Given the two groups of patients are separated in time, some of the improvements may be related to the multi-disciplinary surgical team being further along the learning curve. These limitations should be taken into consideration when interpreting the results.

## CONCLUSION

In this single surgeon series, moving from minimally invasive to totally endoscopic mitral valve surgery has been a positive experience with key patient advantages characterized by smaller incisions, the avoidance of undermining the wound and the absence of rib spreading for improved access. This dataset demonstrated improved patient outcomes, including reduced postoperative bleeding, pain, hospital stay duration and enhanced cosmesis. However, given its inherent limitations and uncontrolled nature, a larger study with long-term follow-up is warranted to validate these findings.

## Data Availability

The data underlying this article will be shared on reasonable request to the corresponding author.

## ABBREVIATIONS

AKI: Acute kidney injury
CPB: Cardiopulmonary bypass
CVA: Cerebrovascular accident
MIMVS: Minimally invasive mitral valve surgery
PFO: Patient foramen ovale
TOE: Transoesophageal echocardiography
